# Enhancement of mesenchymal stem cells’ chondrogenic potential by type II collagen-based bioscaffolds

**DOI:** 10.1007/s11033-023-08461-x

**Published:** 2023-04-28

**Authors:** Zoi Piperigkou, Dimitra Bainantzou, Nadia Makri, Eleni Papachristou, Aglaia Mantsou, Theodora Choli-Papadopoulou, Achilleas D. Theocharis, Nikos K. Karamanos

**Affiliations:** 1 Biochemistry, Biochemical Analysis and Matrix Pathobiology Research Group, Laboratory of Biochemistry, Department of Chemistry, University of Patras, Patras, Greece; 2 Foundation for Research and Technology-Hellas (FORTH)/Institute of Chemical Engineering Sciences (ICE-HT), Patras, Greece; 3 Laboratory of Biochemistry, Department of Chemistry, Aristotle University of Thessaloniki, Thessaloniki, Greece

**Keywords:** Mesenchymal stem cells, Osteoarthritis, Extracellular matrix, Type II collagen, Chondrogenic differentiation, Molecular targeting

## Abstract

**Background:**

Osteoarthritis (OA) is a common degenerative chronic disease accounting for physical pain, tissue stiffness and mobility restriction. Current therapeutic approaches fail to prevent the progression of the disease considering the limited knowledge on OA pathobiology. During OA progression, the extracellular matrix (ECM) of the cartilage is aberrantly remodeled by chondrocytes. Chondrocytes, being the main cell population of the cartilage, participate in cartilage regeneration process. To this end, modern tissue engineering strategies involve the recruitment of mesenchymal stem cells (MSCs) due to their regenerative capacity as to promote chondrocyte self-regeneration.

**Methods and results:**

In the present study, we evaluated the role of type II collagen, as the main matrix macromolecule in the cartilage matrix, to promote chondrogenic differentiation in two MSC in vitro culture systems. The chondrogenic differentiation of human Wharton’s jelly- and dental pulp-derived MSCs was investigated over a 24-day culture period on type II collagen coating to improve the binding affinity of MSCs. Functional assays, demonstrated that type II collagen promoted chondrogenic differentiation in both MSCs tested, which was confirmed through gene and protein analysis of major chondrogenic markers.

**Conclusions:**

Our data support that type II collagen contributes as a natural bioscaffold enhancing chondrogenesis in both MSC models, thus enhancing the commitment of MSC-based therapeutic approaches in regenerative medicine to target OA and bring therapy closer to the clinical use.

**Supplementary Information:**

The online version contains supplementary material available at 10.1007/s11033-023-08461-x.

## Introduction

ECM is a three-dimensional (3D) and well-organized architectural network of macromolecules that functions as a physical scaffold into which cells are embedded [1, 2]. This macromolecular network is comprised by a wide variety of molecules, each one demonstrating distinct structural and functional roles in cells and tissues. The major constituents of ECM are collagens, elastin, proteoglycans (PGs) and glycosaminoglycans (GAGs) and several other glycoproteins, such as matricellular proteins [3]. Through chemical and mechanical signals, the ECM coordinates and regulates many cellular processes, including homeostasis, proliferation, differentiation, morphogenesis, survival, and migration [4].

Hyaline cartilage is mainly located on the articular surfaces of the bones involved in movement and it is composed of abundant ECM and chondrocytes [5]. Chondrocytes are surrounded by a pericellular matrix plentiful in perlecan and type VI collagen, while the surrounding matrix is rich in type II collagen decorated with type IX collagen and small leucine-rich PGs (SLRPs) that participate in collagen fibrillogenesis and provide cartilage integrity and elasticity [6]. Another major matrix components are the large aggrecan-hyaluronan (HA) aggregates folded within type II collagen fibrils and regulate cartilage biomechanical properties. Lacking nerves and blood vessels, low-proliferative chondrocytes have the reparative and regenerative ability of cartilage in cases of injury or disease, such as in OA [7].

The most common degenerative joint disease, OA, is characterized by the progressive disintegration of the cartilage that results in functional and structural alterations in the joint. The inflammatory environment of OA causes the stressed chondrocytes to produce pro-inflammatory cytokines which trigger the secretion of reactive oxygen species (ROS), a disintegrin and metalloproteinase with thrombospondin motifs (ADAMTSs) and matrix metalloproteinases (MMPs). These matrix enzymes promote the degradation of collagens, aggrecan, and aggrecan-HA aggregates, producing aggrecan and bioactive HA fragments that amplify the cartilage inflammation and degradation [3, 8].

In recent years, tissue engineering led to new technological approaches, with the manufacture of artificial biomimetic scaffolds using protein polypeptides, such as type II collagen fibrils and the recruitment of mesenchymal stem cells (MSCs) to regenerate articular cartilage in vitro. Protein polypeptides are very useful in tissue engineering strategies because they can self-assemble and form 3D fibrous networks that can support MSC proliferation and ECM formation [9, 10]. Moreover, MSCs are considered an attractive source of cells in regenerative medicine, as they can be isolated very easily, they have a high migratory and proliferative capacity, and they can differentiate into chondrocytes. Stem cells derived from human permanent third molar pulp (DPSCs) are normally located in the perivascular space of the dental pulp [11] and they have been shown to possess an inherent capacity for multilineage differentiation to osteogenic/odontogenic, myogenic, adipogenic, chondrogenic, angiogenic and neurogenic cell types [12-14]. Their application spans from tissue regeneration (i.e., dentin, bone) to advanced treatment solutions, such as in myocardial infraction, neurodegenerative diseases (i.e., Parkinson’s, Alzheimer’s), diabetes, muscular dystrophy, and liver diseases [15-17]. The cellular functions of DPSCs are affected strongly by the secretion of several growth factors, such as basic fibroblast growth factor (bFGF), transforming growth factor β, (TGF-β), nerve growth factor (NGF), platelet-derived growth factor (PDGF), and bone morphogenetic proteins (BMPs) that upon binding to specific receptors, guide cell differentiation and proliferation [15]. Human Wharton’s jelly MSCs (WJ-MSCs) are a class of stem cells derived from placenta umbilical cord, which are characterized by high differentiative potential, and easy collection process. The use of WJ-MSCs raise no legal or ethical issues since they do not form teratomas while retaining the features of embryonic stem cells. The primitive characteristics of WJ-MSCs make them appealing as candidates of novel therapeutic applications [18, 19]. The distinct features of stem cell populations open new areas for developing advanced targeted for cell-based therapies.

In the light of new research focusing on tissue regeneration, the goal of this study is to evaluate the pathobiology of OA and the impact of matrix reorganization in chondrocyte differentiation and the formation of hyaline cartilage. To this end, WJ- and dental pulp-derived MSCs were assessed for their potential to promote chondrogenic differentiation following coatings on type II collagen. Type II collagen is abundant in the cartilage ECM [3]; thus, the use of type II collagen coatings may provide high affinity binding of MSCs to the defective articular joint.

## Materials and methods

### Cell culture and conditions

Human WJ-MSCs at passage three were isolated and kindly provided by Prof. G. Koliakos and Dr. Eleni Gounari from Biohellenika S.A. MSCs were collected and centrifuged at 750 g for 10 min, resuspended in complete medium [Dulbecco’s Modified Eagle’s Medium (DMEM)] supplemented with 10% fetal bovine serum (FBS), 1% penicillin/streptomycin and 10 μg/ml gentamicin sulfate and routinely cultured in a humidified 95% air/5% CO_2_ incubator at 37 °C [20]. DPSCs at passage 2 were kindly provided by Ass. Prof. A. Bakopoulou (School of Dentistry, Aristotle University of Thessaloniki), and were collected by the enzymatic dissociation method described in Bakopoulou et al. [21]. DPSCs were cultured in alpha-MEM (Minimum Essential Media) culture medium (Invitrogen), supplemented with 15% FBS,100 mM L-ascorbic acid phosphate (Sigma–Aldrich, Taufkirchen, Germany), 100 mg/ml streptomycin, 0.25 mg/ml amphotericin B and (Invitrogen) 100 units/ml penicillin.

Cells were incubated at 37 °C in 5% CO_2_, and after reaching 80% of confluency, both WJ- and dental pulp-derived MSC cultures (passage 2–6) were harvested by trypsinization with 0.05% (w/v) trypsin in PBS containing 0.02% (w/v) Na_2_EDTA and centrifuged at 750 g for 3 min. The cell pellet was resuspended in complete medium, and cells were counted prior to seeding in the respective cell culture plate for each experimental procedure described below. Wells of the respective plates either have been coated with type II collagen at working concentration 1 mg/ml in 0.25% (v/v) CH_3_COOH (0.3 mg/cm^2^) or not (control samples). Following solidification of type II collagen for 1 h at 37 °C, MSCs were seeded on top of the formed collagen coating at a density of 5,000 cells/cm^2^. Every 48 h the culture medium was renewed.

### Chemicals and reagents

Dulbecco’s modified eagle medium (DMEM), fetal bovine serum (FBS), penicillin/streptomycin and gentamicin were all obtained from Biosera (Nuaillé, France). Type II collagen purchased from Sigma Aldrich (St. Louis, MO, USA). All other chemicals used were of the best commercially available grade.

### Alcian blue staining

Alcian blue staining may detect GAG chains in the ECM of chondrocytes. To prepare Alcian blue solution, 1 g Alcian blue (SERVA Electrophoresis GmbH) was dissolved in 100 ml 3% (v/v) CH_3_COOH. Human WJ-MSCs and DPSCs at passage 5 were seeded in 24-well cell culture plates (5,000 cells/well) in complete medium, in the presence or in the absence of 1 mg/ml type II collagen. MSCs were cultured for 14 and 24 days in DMEM 2% FBS. Following the treatment period, culture medium was removed, cell monolayers were washed once with PBS 1X  and fixed in 4% (w/v) paraformaldehyde (AppliChem GmbH) in PBS 1X  for 30 min at room temperature. Cells were washed three times with PBS 1X  and stained with Alcian blue staining solution overnight in plate shaker at room temperature. The staining solution was carefully discarded, and wells are washed twice with 3% (v/v) CH_3_COOH and once with distilled H_2_O. Images of WJ-MSCs were captured using an inverted OLYMPUS CKX41 microscope equipped with a CMOS color digital camera (SC30) at 10 × magnification. Images of DPSCs were captured using a Nikon DS-Fi3 microscope camera.

### Cell viability assay

Wharton’s jelly MSCs and DPSCs at passage five were seeded in 48-well plates containing type II collagen at a density of 3,200 cells/well and then the cells were incubated in DMEM supplemented with 2% FBS for 3, 7, 10, 14 and 24 days. Medium was replaced every two days following one wash with PBS 1X. Premix WST-1 (water-soluble tetrazolium salt) Cell Proliferation Assay System (Takara Bio Inc., Japan) was added after the treatment period at a ratio 1:10 for 1-3 h depending on cell density. The assay is based on the reduction of WST-1 by viable cells, producing a soluble formazan salt absorbing at 450 nm (reference wavelength at 650 nm). Following incubation, the optical density of each well was measured at 450 nm with a TECAN spectrophotometer.

### RNA isolation, reverse transcription and real-time qPCR analysis

Total cellular RNA from Whartons’ jelly MSCs and DPSCs (5,000cells/cm^2^) was isolated using NucleoSpin RNA II Kit (Macherey–Nagel, Duren, Germany) after treatment in the absence or presence of type II collagen (1 mg/ml) for 24 days. The amount of isolated RNA was quantified by measuring its absorbance at 260 nm. Total RNA was reverse transcribed using the PrimeScript 1st strand cDNA synthesis kit perfect real time (Takara Bio Inc., Japan) and the Finnzymes RobusT^TM^I kit, for Wharton’s jelly MSCs and DPSCs, respectively. Real-time qPCR analysis was conducted in 20 μl reaction mixture, according to manufacturer’s instructions (RobusT™I kit, Finnzymes; KAPA Taq ReadyMix DNA Polymerase, KAPA BIOSYSTEMS). The amplification was performed utilizing Rotor Gene Q (Qiagen, USA) and Step One Plus thermal cycler (Applied Biosystems, USA). All reactions were performed in triplicates and a standard curve was always included for each pair of primers for assay validation. In addition, a melting curve analysis was always performed for detecting the SYBR Green-based objective amplicon. To provide quantification, the point of product accumulation in the early logarithmic phase of the amplification plot was defined by assigning a fluorescence threshold above the background, defined as the threshold cycle (Ct) number. Relative expression of different gene transcripts was calculated by the ΔΔCt method. The Ct value of any gene of interest was normalized to the Ct of the normalizer (GAPDH). Fold changes (arbitrary units) were determined as 2^−ΔΔCt^. Primer sequences of the tested genes are presented in Table 1. All primers were purchased from Eurofins Genomics (Ebersberg, Germany).

**Table 1:** List of real-time PCR primer sequences utilized in the study

Gene of interest	Forward primer	Reverse primer	T_an_
Aggrecan	5′-TCGAGGACAGCGAGGCC-3′	5′-TCGAGGGTGTAGCGTGTAGAGA-3′	56
SOX9	5′-CCCATGTGGAAGGCAGATG-3’	5′-TTCTGAGAGGCACAGGTGACA-3′	60
RUNX2	5′-GTACAGCTTTAAGGATTCCCTCAATTC-3′	5′-TTGCTAATGCTTCGTGTTTCCA-3′	60
COLII	5′-GGCAATAGCAGGTTCACGTACA-3′	5′-CGATAACAGTCTTGCCCCACTT-3′	57
COLX	5′-ACAGGAATGCCTGTGTCTGCTTTACT-3′	5′-CATTGGGAAGCTGGAGCCACACCTGGTC-3′	60
Osteocalcin	5′-ACACTCCTCGCCCTATTG-3′	5′-GATGTGGTCAGCCAACTC-3′	60
ALP	5′-CCTGGCTTTCTCGTCACTCTCA-3′	5′-CCTGGCTTTCTCGTCACTCTCA-3′	60
MMP1	5′-CCT CGC TGG GAG CAA ACA-3′	5′-TTG GCA AAT CTG GCG TGT AA-3′	60
MMP2	5′-CGT CTG TCC CAG GAT GAC ATC-3′	5′-ATG TCA GGA GAG GCC CCA TA-3′	62
MMP7	5′-GCTGGCTCATGCCTTTGC-3′	5′’-TCCTCATCGAAGTGAGCATCTC	60
MMP9	5′-TTCCAGTACCGAGAGAAAGCCTAT-3′	5′-GGTCACGTAGCCCACTTGGT-3′	60
MMP13	5′-AAGGAGCATGGCGACTTCT-3′	5′-TGGCCCAGGAGGAAAAGC-3′	57
TIMP1	5′-CGCTGACATCCGGTTCGT-3′	5′-TGTGGAAGTATCCGCAGACACT-3′	60
TIMP2	5′-GGGCACCAGGCCAAGTT-3′	5′-CGCACAGGAGCCATCACT-3′	60
GAPDH	5′-AGGCTGTTGTCATACTTCTCAT-3′	5′-GGAGTCCACTGGCGTCTT-3′	60

### Western blot analysis

Cell monolayers of MSCs treated with type II collagen for 24 days were washed with cold PBS and lysed with Lysis Buffer: 25 mM Hepes, pH 7.5, 150 mM NaCl, 5 mM EDTA, 10% (v/v) glycerol, 1% (v/v) Triton X-100, containing protease inhibitor cocktail (#20–201 Chemicon, Millipore, CA) and 0.5 mM sodium orthovanadate (S6508, Sigma-Aldrich, Inc). Samples were reduced with β-mercaptoethanol in Laemmli sample buffer, separated by SDS-PAGE in 12% poly-acrylamide gels and transferred to polyvinylidene difluoride (PVDF) membranes (Macherey Nagel, Germany). The membranes were blocked in 5% (w/v) non-fat dry milk in Tris-buffered saline pH 7.4 containing 0.05% Tween-20 (TBS-T) for 2 h at room temperature and were then incubated with primary antibodies for 16 h at 4 °C. After three washes in TBS-T, membranes were further incubated with peroxidase-conjugated secondary goat anti-rabbit IgG (A0545, Sigma-Aldrich, Inc) or anti-mouse IgG (A4416, Sigma-Aldrich, Inc) for 90 min at room temperature. Detection of the immunoreactive proteins was performed by chemiluminescence horseradish peroxidase substrate Super Signal (Pierce, Thermoscientific), according to the manufacturer’s instructions. Primary antibodies used in immunoblotting include p-ERK1/2 (9101, Cell Signaling Technology, dilution 1:1000), total ERK1/2 (9102, Cell Signaling Technology, dilution 1:1000), p-Akt (4060, Cell Signaling Technology, dilution 1:1000), GAPDH (G9545, Sigma-Aldrich Inc., dilution 1:5000), and α-tubulin (T9026, Sigma-Aldrich Inc., dilution 1:7500).

### Statistical analysis

Reported values are expressed as mean ± standard deviation (SD) of experiments in triplicate. Three independent biological samples have been used in each experimental set. Statistically significant differences were evaluated using the analysis of variance (two-way ANOVA) test and were considered statistically significant at the level of at least p ≤ 0.05. Statistical analysis and graphs were made using GraphPad Prism 8.2.1. Software.

## Results

The major deficiency in MSCs application in clinical practice focuses on the limited survival period following injection to the affected tissue. To solve this limitation, higher affinity binding towards the targeted tissue following injections with MSCs should be achieved to create an environment for MSCs to coalesce, since cell communication is paramount for cell survival. To this end, the ability of type II collagen to promote chondrogenic differentiation in MSCs in favor of cartilage regeneration was investigated. Human umbilical cord Wharton’s jelly (*substantia gelatinea funiculi umbilicalis*) and dental pulp are both valuable sources of multipotent MSCs with high chondrogenic potential [22-25]. Moreover, multiple studies have demonstrated that DPSCs possess significant regenerative and anti-inflammatory properties. The functional properties of DPSCs, such as cell attachment, proliferation, differentiation, and angiogenesis have been found to be affected by a variety of biomaterials (i.e., collagen scaffolds, hydrogels, decellularized matrices etc.) [26-28].

### Type II collagen increases the long-term viability of MSCs

**Fig. 1:**
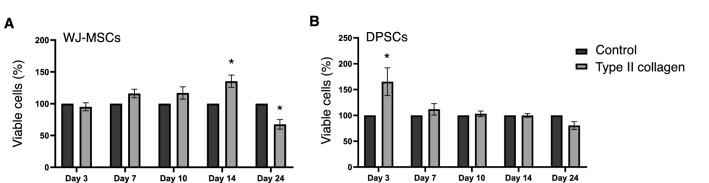
Type II collagen coating effects on cell viability of MSCs. The viability of Wharton’s jelly MSCs (**A**) and DPSCs (**B**) was evaluated with MTT assay at 3, 7, 10, 14 and 24 days of culture in the absence or presence of type II collagen. The data are presented as the mean ± SD values (n = 3). Asterisk (*) indicates statistically significant differences (p ≤ 0.05) compared to control cells of day 3. *DPSCs* dental pulp mesenchymal stem cells, *MSCs* mesenchymal stem cells, *MTT* (3-[4,5-dimethylthiazol-2-yl]-2,5 diphenyl tetrazolium bromide)

As depicted in Fig. 1, type II collagen (1 mg/ml) coating enhances cell viability of both WJ-MSCs (Fig. 1A) and DPSCs (Fig. 1B) until day 14 of treatment, suggesting that this fibrillar protein demonstrates no significant cytotoxicity in both MSCs cultures and it acts favorably for cell growth. Notably, our results showed that type II collagen demonstrates increased biocompatibility in both MSCs types. WJ-MSCs showed 20–50% increased cell growth when cultured on type II collagen, while the maximum effect was observed at day 14 (Fig. 1A). This effect was observed at day 3 of DPSCs culture where type II collagen resulted in 60% increase in the viability of these cells (Fig. 1B). Intriguingly, both MSC lines showed decreased cell viability at day 24 of culture which could be correlated with the increased chondrogenic differentiation. This interesting finding prompted us to further confirm the chondrogenic potential of MSCs on type II collagen coatings.

### Type II collagen controls the chondrogenic potential of MSCs

**Fig. 2:**
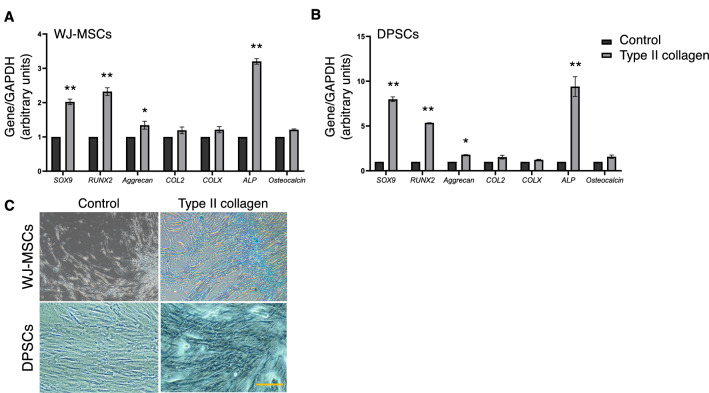
Type II collagen reinforces the expression of chondrogenic markers in MSCs. Type II collagen coating for 24 days markedly increases the mRNA levels of *SOX9*, *RUNX2*, *aggrecan* and *ALP* in WJ-MSCs (**A**) and DPSCs (**B**). The data are presented as the mean ± SD values (n = 3). Asterisks (*), (**) indicate statistically significant differences (p ≤ 0.05 and p ≤ 0.01, respectively) compared to control cells. (**C**) GAG accumulation during chondrogenic differentiation assessed by Alcian blue staining and phase-contrast microscopy. Photographs of monolayer expanded WJ-MSCs and DPSCs cultured for 24 days in the absence or presence of type II collagen (1 mg/ml). Scale bar, 20 μm. *ALP* alkaline phosphatase, *COL2* type II collagen, *COLX* type X collagen; *DPSCs* dental pulp mesenchymal stem cells, *WJ-MSCs* Wharton’s jelly mesenchymal stem cells

The extend of chondrogenesis in WJ-MSCs and DPSCs was evaluated by estimating the expression levels of chondrogenic transcription factors and matrix components as well as the extend of GAG synthesis through Alcian blue staining. Real-time PCR analysis applied to quantify the mRNA levels of a variety of genes related to chondrogenic differentiation towards the formation of hyaline cartilage. As shown in Fig. 2, coating of both WJ-MSCs and DPSCs in type II collagen increased the mRNA expression levels of major chondrogenic transcription factors, PGs, collagens, matrix proteins, and enzymes.

Specifically, 24 days of WJ-MSCs culture on type II collagen markedly increased the expression of typical chondrogenic transcription markers, including *SOX9* and *RUNX2* (2- and 2.5-fold, respectively, compared to control cells, Fig. 2A). Moreover, *aggrecan*, the key multimodular PG expressed by chondrocytes [29], demonstrated a significant increase under these conditions as compared to control cells. The expression of major hypertrophy-associated genes, such as *type X collagen* and *ALP*, was also evaluated. The mRNA levels of *COL2* and the hypertrophic marker *COLX* followed the same trend and found only slightly increased (20%) in respect to untreated cells. Notably, a threefold increase in the mRNA levels of *ALP* was observed. This can be correlated with the view that type II collagen coating induced chondrogenesis in WJ-MSCs and lead to a hypertrophic phenotype in a 24-days culture. Notably, such increase in *ALP* has not been observed in these MSCs following a 14-day culture on type II collagen coating (data not shown). Moreover, osteocalcin has been reported to affect cartilage development by regulating normal processes of endochondral bone formation [30]. WJ-MSCs demonstrated a slight increase in the mRNA levels of *osteocalcin* (0.25-fold compared to control cells) supporting the view that WJ-MSCs do not expand the differentiation process to osteoblasts but remain in the chondrocyte state enhancing the ability of type II collagen for controlled chondrogenesis.

Similar pattern is observed for DPSCs when cultured on type II collagen coating in the same conditions (Fig. 2B). Specifically, after 24 days of DPSCs culture in the presence of type II collagen, the mRNA levels of typical chondrogenic transcription factors, *SOX9* and *RUNX2*, demonstrated a robust increase, eight- and fivefold, respectively, as compared to the control group. Intriguingly, DPSCs displayed stronger increase compared to that observed in WJ-MSCs. Moreover, type II collagen induced a 2-fold increase in *aggrecan* mRNA levels. Notably, DPSCs displayed a ninefold statistically significant increase in *ALP* levels, supporting the view of hypertrophic chondrocytes development following type II collagen coating for 24 days. Although, no statistically significant changes in the mRNA levels of *COL2*, *COLX* and *osteocalcin* were observed.

Taking into consideration the expression levels of typical chondrogenic markers, we conclude that type II collagen demonstrated a stronger effect in inducing the chondrogenic potential of DPSCs rather than in WJ-MSCs; however both MSC lines exhibited increased chondrogenic differentiation via the increased expression of major chondrogenic markers. These results were confirmed by Alcian blue staining that certified the enhanced chondrogenic differentiation in both DPSCs and WJ-MSCs (Fig. 2C) cultured in type II collagen. As shown in Fig. 2C, Alcian blue stained the sulfated PGs at day 24 in both MSC lines, providing evidence for chondrogenic differentiation in both DPSCs and WJ-MSCs. Notably, chondrocytes can be observed in type II collagen-cultured WJ-MSCs and DPSCs (Fig. 2C).

### The expression of major MMPs and TIMPs is affected by type II collagen in MSCs

**Fig. 3:**
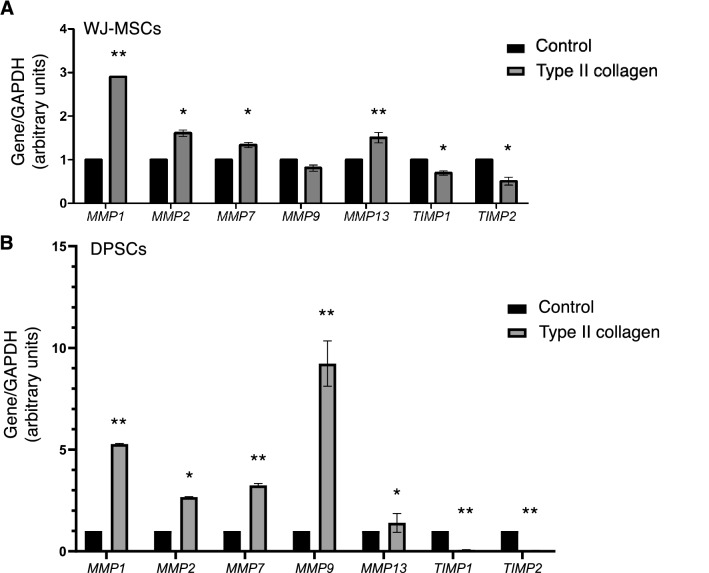
Type II collagen induces chondrogenic differentiation MSCs by mediating the expression of MMPs and TIMPs. Type II collagen coating for 24 days markedly affects the mRNA levels of *mmp1, mmp2, mmp7, mmp9, mmp13, timp1* and *timp2* in WJ-MSCs (**A**) and DPSCs (**B**). The data are presented as the mean ± SD values (n = 3). Asterisks (*), (**) indicate statistically significant differences (p ≤ 0.05 and p ≤ 0.01, respectively) compared to control cells. *DPSCs* dental pulp mesenchymal stem cells, *mmp* matrix metalloproteinase gene, *timp* endogenous MMP inhibitor gene, *WJ-MSCs* Wharton’s jelly mesenchymal stem cells

After the 24-day culture of WJ-MSCs in the presence of type II collagen, these cells demonstrated a statistically significant increase in the expression levels of *mmp1*, *mmp2*, *mmp7*, *mmp13* at the level of 3-, 1.5-, 1.2-, and 1.5-fold, respectively (Fig. 3A). Notably, *mmp9* levels remained unaffected by type II collagen. These data suggest the crucial role of these MMPs during the chondrogenic differentiation of WJ-MSCs mediated by type II collagen. Furthermore, type II collagen significantly reduced the mRNA levels of *timp1* and *timp2* that can be correlated with the actions of TIMPs on MMP activation.

Intriguingly, the impact of type II collagen on the expression of MMPs and TIMPs was more prominent in DPSCs 24-day cultures. Specifically, as shown in Fig. 3B, type II collagen resulted in a fivefold increase in *mmp1* levels, twofold increase in *mmp2* and *mmp7*, where as its effect on *mmp9* expression was the striking ninefold upregulation. The increased activity of MMP-9 has been correlated with ECM accumulation in the cartilaginous nodules, during the growth of the articular cartilage in the early stages of chondrogenic differentiation [31]. Type II collagen also resulted in a statistically significant induction in *mmp13* levels, whereas it diminished the expression of *timp1* and *timp2*. Overall, this data suggests the great potential of type II collagen in regulated the expression of major MMPs and TIMPs that in turn mediate ECM turnover during chondrogenic differentiation in both MSC cultures investigated in the present study.

### Type II collagen mediates the activation of ERK1/2 and PI3K/Akt in MSCs

Chondrogenesis is a well-orchestrated differentiation process where, as cartilage enlarges, articular chondrocytes reorganize specific signaling cascades orchestrated by protein kinases and downstream molecules. The mitogen-activated protein kinase (MAPK) group of signal transduction pathways transmits extracellular signals that stimulate mitogenic and stress responses [32]. Moreover, the hypoxia-dependent protection from apoptosis is related to the activation of phosphoinositide-3 kinase (PI3K) and its major downstream molecule, Akt, enhances chondrocyte proliferation and inhibited hypertrophic differentiation in chondrogenesis [33].

**Fig. 4:**
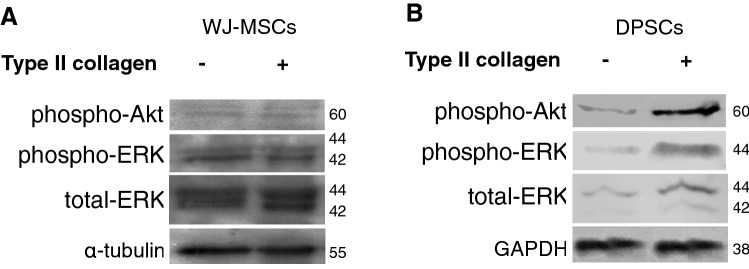
Effect of type II collagen on the activation of ERK1/2 and PI3K/Akt signaling cascades in MSCs. Protein lysates following 24-day culture of WJ-MSCs (**A**) and DPSCs (**B**) were assayed with western blotting analysis for phospho-ERK1/2, and phospho-Akt. GAPDH and α-tubulin were used as normalizers of DPSCs and WJ-MSCs, respectively. Blots were cropped in the figure, but the gels were run under the same experimental conditions. Three experiments were conducted at different times for each MSC line and representative blots are shown

Our results showed that type II collagen coating induced the phosphorylation of extracellular signal-regulated kinase 1/2 (ERK1/2) and PI3K/Akt in both WJ-MSCs and DPSCs (Fig. 4A and B).

## Discussion

During the last decades, the tremendous interest of cell-based therapies for OA has led to continued research in the area of regenerative medicine as to create prosperous therapeutic approaches for healing tissue pathologies and orthopaedic fractures [34, 35]. Due to the decreased metabolic activity and the avascular structure of chondrocytes, cartilage does not proceed to self-repair mechanisms, thus making the effective treatment of cartilage injury a major problem for clinicians. To this end, the drawbacks of effective for cartilage injury treatments have led to the advancement of novel regenerative medical therapies. Cell populations are characterized by enormous therapeutic potential, since they provide tissue-specific mechanisms that chemical drug formulations cannot mimic.

MSCs largely drive tissue regeneration due to their capacity to directionally differentiate and proliferate [36]. The outstanding ability of MSCs to undergo chondrogenic differentiation following extensive expansion in vitro and stimulation with bioscaffolds in 3D systems, has made MSC-based therapeutic approaches a potential alternative strategy to manage cartilage repair [37]. However, these approaches have limited success, mainly due to deficient cell localization, and survival rates at the site of injection [38].

During chondrogenic differentiation in MSCs, the deposited ECM acts as the protector of the transplanted cells. MSCs take advantage of the cell-cell and cell-ECM interactions at the targeted repair areas, driving the release of growth factors, chemokines and cytokines creating an appropriate regenerative microenvironment for cartilage repair [39, 40]. Type II collagen is among the major biomolecules of the hyaline cartilage and has a key role in maintaining chondrocyte function. To this end, we evaluated the potential of MSCs combination with exogenous stimuli such as type II collagen in advancing cartilage regeneration. 2D cultures of WJ-MSCs and DPSCs on top of thin layers of type II collagen coatings were assessed regarding their chondrogenic differentiation potential. The formation of collagen fibers (Supplementary Figure) was achieved in 2D cultures of MSCs. Chondrogenic differentiation of WJ-MSCs and DPSCs was at first confirmed by Alcian blue staining for GAG matrix synthesis that has been further confirmed in molecular level.

The cartilage-specific transcription factor, *SOX9,* is the essential transcription factor of the chondrogenic lineage, directly regulating the expression of chondrogenic genes, such as *type II collagen* [41, 42]. Chondrocytes generated from differentiated MSCs express typical genes as the native chondrocytes, for instance, *type II collagen* and *aggrecan*. Aggrecan, the major PG found in hyaline cartilage, is the major factor to create the cartilage’s fixed negative charge due to its GAG content [43]. Moreover, HA-aggrecan aggregates are captured within type II collagen fibrils forming a complex structure that gives cartilage its ability to absorb throbs. The anchorage of aggrecan to the cell surface is due to the binding of aggrecan to HA either via the multivalent binding to CD44 or via a HA synthase (HAS) [29]. The activation of *aggrecan* in both WJ-MSCs and in DPSCs that we confirmed in our study, may be correlated with increased aggrecanase activity of ADAMTS-4 and -5 that has been reported in several studies [8]. Moreover, it has been established that *SOX9* enhances *aggrecan* activity [44], which was also confirmed by our results, showing increased *aggrecan* expression in both WJ-MSCs and in DPSCs followed by increased *SOX9* expression in MSCs in the presence of type II collagen.

It is well established that MSCs express hypertrophic markers after chondrogenic induction in vitro and in vivo, hindering their clinical application for cartilage tissue regeneration. One major factor that controls cell fate in chondrocytes is phosphate (Pi). Pi is a critical signaling molecule and its cell response is orchestrated by matrix composition and the activity of phosphatases, such as alkaline phosphatase (ALP). ALP cleaves PPi to release Pi and its expression is higher in the maturing zone of chondrogenic differentiation [45]. In the present study, we identified hypertrophy-associated genes, as *COLX*, *ALP* and *mmp13*, following type II collagen coating of cultures of both WJ-MSCs and DPSCs. These macromolecules are expressed after the activation of the transcription factor *RUNX2* by the hypertrophic chondrocytes, in order to remodel the ECM for the subsequent process of endochondral ossification. Recent data support the view that cartilage and bone are in molecular crosstalk over the calcified tissue barrier via several signaling cascades, such as those activated by BMPs and Wnts [30, 46]. In OA, chondrocytes can differentiate to a hypertrophic phenotype characterized by type X collagen. Our results showed that human WJ-MSCs express high levels of *RUNX2, ALP*, *mmp13* and *osteocalcin* supporting our view that type II collagen promotes chondrogenic potential of MSCs and that hypertrophic populations of differentiated MSCs exist following 24-day culture.

MMPs are crucial in several developmental processes, such as tissue remodeling, cell migration, adhesion, invasion, proliferation, and apoptosis [47, 48]. ECM remodeling is a crucial process for chondrocyte progenitor cells to undergo differentiation during skeletal formation, followed by the increase in collagen concentration, yielding increased matrix production [49]. It is well established that MSCs interact with secreted MMPs, thus mediating the pericellular MMP functions [31]. The fate of MSCs is also regulated by specific MMPs and their inhibitors, TIMPs, and is associated with a distinct cell lineage. It is well established that TIMPs play key roles in the balance of ECM turnover. Notably, *timp1* knockdown enhances MSCs proliferation, which also suggests a negative effect of TIMP1 on proliferation [50]. Recent reports suggest the integration of MMPs in the functional properties of MSCs including proliferation, migration, differentiation, and angiogenesis [36]. However, this interplay is not fully understood and warrants further investigation. In particular, MMP7 and MMP14 can activate other MMPs such as pro-MMP1, and pro-MMP13, respectively [51]. On the other hand, MMP2 activity is increased during the differentiation of MSCs from human adipose tissue into chondrocytes [52]. Finally, MMP9 function is reported to be involved in the final stages of chondrogenesis and its expression increases mainly during endochondral ossification, with the aim of matrix remodeling for bone formation and angiogenesis, while its deficiency has been found to lead to delayed bone development [52]. Our results pinpointed that type II collagen in WJ-MSCs and in DPSCs resulted in profound increase in *mmp1*, *mmp2* and *mmp7*, while *timp1* and *timp2* expression was downregulated. Notably, in DPSCs cultured on type II collagen coating, the expression of the gelatinase MMP9 was excessively increased, which may be correlated with MMP9 capacity to degrade type II, IX, and XI collagen, activating pro-collagenase I and releasing angiogenic factors in the hypertrophic cartilage ECM [52].

Overall, the novel findings of the present study highlighted the key role of type II collagen in the long-term growth and profound chondrogenic potential of WJ-MSCs and DPSCs through the recruitment of chondrogenic transcription factors, GAG synthesis, major matrix components, MMPs/TIMPs enzymatic actions and the activation of kinase-dependent signaling cascades regulating cell proliferation.

## Conclusion

The ECM is a dynamic and complex structural and functional network capable of mediating the MSC differentiation under certain conditions. The present study assessed the impact of type II collagen coating on the bioavailability and chondrogenic potential of WJ-MSCs and DPSCs. In general, data showed that type II collagen promoted chondrogenic induction in long time points in both human MSC models in vitro. Overall, we highlighted the significance of type II collagen in the growth, matrix reorganization and chondrogenic differentiation of human MSCs that has to be considered for future cell-based therapeutic approaches being developed in favor of degenerative pathologies.

## Supplementary Information

Below is the link to the electronic supplementary material.

Supplementary file1 (DOCX 431 KB)

## Abbreviations

ADAMTS: A disintegrin and metalloproteinase with thrombospondin motifs
bFGF: Basic fibroblast growth factor
BMPs: Bone morphogenetic proteins
DPSCs: Dental pulp stem cells
ECM: Extracellular matrix
GAGs: Glycosaminoglycans
HA: Hyaluronan
MMPs: Matrix metalloproteinases
MSCs: Mesenchymal stem cells
NGF: Nerve growth factor
OA: Osteoarthritis
PDGF: Platelet-derived growth factor
PGs: Proteoglycans
ROS: Reactive oxygen species
SLRPs: Small leucine-rich proteoglycans
TIMPs: Endogenous inhibitors of MMPs
WJ-MSCs: Wharton’s jelly MSC
