# Supplementary material for: Enhancement of mesenchymal stem cells’ chondrogenic potential by type II collagen-based bioscaffolds
Source: Mol Biol Rep. 2023 Apr 28;50(6):5125–35. doi: 10.1007/s11033-023-08461-x (PMC10209287; doi:10.1007/s11033-023-08461-x)
Supplement: Supplementary file 1 — Supplementary file1 (DOCX 431 KB) [file 11033_2023_8461_MOESM1_ESM.docx]

**Enhancement of mesenchymal stem cells’ chondrogenic potential by type II collagen-based bioscaffolds**

Zoi Piperigkou^1,2^, Dimitra Bainantzou^1^, Nadia Makri^1^, Eleni Papachristou^3^, Aglaia Mantsou^3^, Theodora Choli-Papadopoulou^3^, Achilleas D. Theocharis^1,*^, Nikos K. Karamanos^1,2,*^

^1^Biochemistry, Biochemical Analysis and Matrix Pathobiology Research Group, Laboratory of Biochemistry, Department of Chemistry, University of Patras, Greece.

^2^Foundation for Research and Technology-Hellas (FORTH)/Institute of Chemical Engineering Sciences (ICE-HT), Patras, Greece.

^3^Laboratory of Biochemistry, Department of Chemistry, Aristotle University of Thessaloniki, Greece.

**Supplementary Figure 1.**


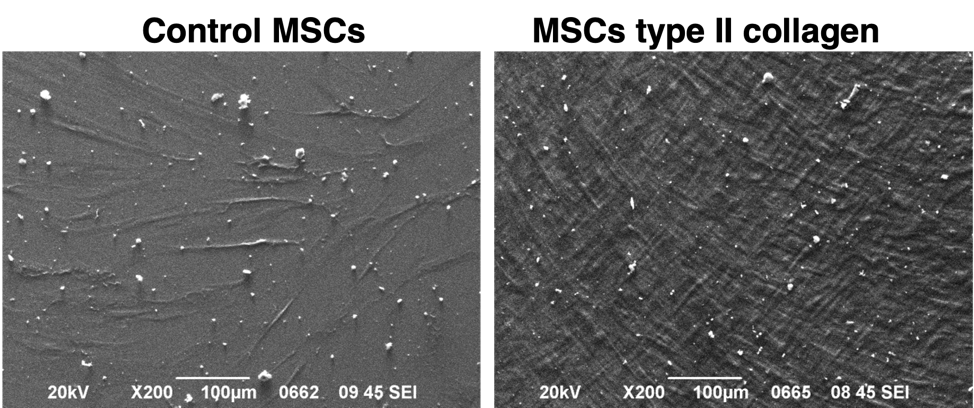


**Supplementary Figure Legend.** Scanning electron microscopy (SEM) images of dental pulp-derived mesenchymal stem cells cultured in plastic culture flasks (control) and in flasks coated with type II collagen, where the collagen fibers can be observed.
